# Relationship between perception of body image on obesity and smoking status by age group in women: Findings of a seven-year Korean National Survey

**DOI:** 10.18332/tid/194098

**Published:** 2024-10-25

**Authors:** Hye Jung Hwang, Youngmee Kim, Won-Kyung Cho

**Affiliations:** 1 Department of Nursing, Chung-Ang University Hospital, Gyeonggi-do, Republic of Korea; 2 Red Cross College of Nursing, Chung-Ang University, Seoul, Republic of Korea; 3 International Healthcare Center, Asan Medical Center, University of Ulsan, Seoul, Republic of Korea; 4 Pulmonary and Critical Care Medicine, Asan Medical Center, University of Ulsan College of Medicine, Seoul, Republic of Korea

**Keywords:** body image perception, obesity, Korean, smoking, women

## Abstract

**INTRODUCTION:**

This descriptive study examined the relationship between body image perception and smoking status among women aged 19–64 years in Korea, using data from the Korea National Health and Nutrition Examination Survey (KNHANES).

**METHODS:**

This study is a secondary analysis of data gathered from the KNHANES between 2014 and 2020, encompassing 12515 women aged 19–64 years. The final study group consisted of 742 current smokers (CS), 132 hidden smokers (HS), and 11641 non-smokers (NS). Hidden smokers were defined as participants who reported being non-smokers but had urine cotinine levels >50 ng/mL. The participants were divided into three age categories: 19–29, 30–49, and 50–64 years. A multiple logistic regression analysis was used to assess the relationship between body image perception and smoking status, by age group.

**RESULTS:**

Baseline statistics indicated that smokers (both CS and HS) generally had a lower socioeconomic status across all age groups. The highest rate of obesity perception was observed in the CS group, followed by the HS group, in both groups of women aged 19–29 and 30–49 years. However, only women aged 19–29 years in the CS group were more likely to perceive themselves as obese than those in the NS group (AOR=2.60; 95% CI: 1.49–4.52; p=0.001). Furthermore, factors such as current smoking status (AOR=2.32; 95% CI: 1.28–4.23; p=0.006), higher body mass index (AOR=2.95; 95% CI: 2.59–3.37; p<0.001), and perceived health status as poor (AOR=3.82; 95% CI: 2.11–6.92; p<0.001), significantly influenced the perception of obesity in this age group.

**CONCLUSIONS:**

This study identified a notable relationship between obesity perception and smoking among women aged 19–29 years only. These findings suggest that interventions aimed at weight reduction or modifying the perception of obesity, could potentially aid smoking cessation efforts in young women.

## INTRODUCTION

Obesity and smoking are global health issues, and they are also significant concerns in South Korea. Using a body mass index (BMI) of ≥25 kg/m^2^ as the definition of obesity in adults aged ≥19 years, the prevalence of obesity was 37.2% in 2022^1^. Additionally, the smoking rate among Koreans aged ≥19 years was 16.9% in 2022^1^. Is there any relationship between the two? Many people seem to believe that smoking can aid in weight loss. Consequently, numerous studies in the past have investigated the impact of smoking on body weight, and there have been reports suggesting such a possibility^2-4^. However, the association between smoking and weight loss remains debatable^5,6^. Nonetheless, the most widely accepted aspect of the relationship between body weight and smoking, with substantial evidence, is that quitting smoking leads to weight gain, at least temporarily^2,7-9^.

Unsurprisingly, studies have shown that a significant proportion of the population uses smoking as a mechanism to manage weight or resist quitting due to concerns about weight gain. These smoking behaviors for weight control have been mostly studied and are most evident among female adolescents^10-13^. These studies imply that the perception of being overweight, including misperception, can considerably influence the initiation and continuation of smoking, especially among young females. This raises an intriguing question about whether perceptions of obesity also affect smoking behaviors in other age groups. We expanded the target population to include all adult women to address this. Using the Korean national survey, we divided participants into three age groups to investigate the association between perception of body image type and smoking status.

## METHODS

### Study design and ethical considerations

This study is a secondary analysis of data collected from the Korea National Health and Nutrition Examination Survey (KNHANES), conducted between 2014 and 2020. The KNHANES, overseen by the Korea Disease Control and Prevention Agency (KDCA), is an ongoing, nationwide, annual, population-based survey. It aims to comprehensively explore the Korean population’s health status, lifestyle choices, and dietary patterns^14^. The survey comprises three main components: health interviews, health assessments, and nutritional enquiries. Trained medical personnel and interviewers conducted health interviews and assessments at a mobile examination center.

Data were retrieved through a designated registration process from the official KNHANES website (https://knhanes.kdca.go.kr/knhanes/eng/index.do)^14^. Prior to participation, written consent was obtained from all participants in the study in accordance with the Declaration of Helsinki. The Korea Disease Control and Prevention Agency’s Institutional Review Board (KDCA IRB) meticulously reviewed and approved the KNHANES survey (approval numbers: 2013-12EXP-03-5C, 2018-01-03-P-A, 2018-01-03-C-A, and 2018-01-03-2C-A).

### Participants

**Figure 1:**
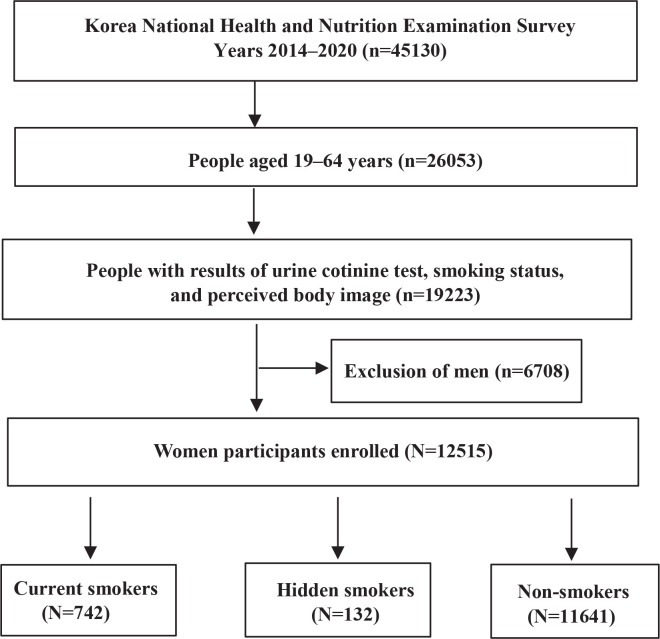
Flow diagram depicting participant selection and the number of participants

Of the 54668 individuals screened, this study enrolled 12515 women aged 19–64 years with data on urine cotinine, perceived body image perception, and smoking status. The final study included 742 current smokers, 132 hidden smokers, and 11641 non-smokers (Figure 1).

### Definition and measurement of important variables

Participants were classified into three groups: current smokers, hidden smokers, and non-smokers (hereafter referred to as CS, HS, and NS, respectively). First, the term ‘current smokers’ described participants who met the following three criteria at the time of participation: 1) had smoked at least 100 cigarettes to date; 2) were still smoking; and 3) had urine cotinine levels of >50 ng/mL. Urine cotinine levels were considered reliable biomarkers for distinguishing between NS and CS^15^. Second, the term ‘hidden smokers’ described participants who reported having smoked in the past but not currently yet had urine cotinine levels of >50 ng/mL. Finally, the term ‘non-smokers’ described participants who reported not currently smoking and had urine cotinine levels of ≤50 ng/mL^16,17^. Data were analyzed based on age, and the participants were grouped into three age groups: 19–29, 30–49, and 50–64 years.

Perceived body image was defined based on the subjective perception of the participants’ own body image. This study grouped categories, such as ‘very thin’ or ‘slightly thin’, as ‘thin’, while ‘normal’ remained a separate category. Additionally, categories, such as ‘slightly obese’ and ‘very obese’, were consolidated into the ‘obese’ category for analysis.

Other variables included in this study were abdominal obesity, defined as a waist circumference of ≥85 cm^18^; perceived health status, referring to an individual’s subjective assessment of their overall health, which is categorized as very good/good, fair, or poor/very poor; and perceived psychological stress, characterized by moderate-to-severe daily stress.

The definitions of other important variables are as follows: trauma history was defined as the occurrence of at least one accident or instance of intoxication requiring hospitalization and/or emergency room treatment within the preceding year. Health behaviors included frequency of alcohol consumption, exercise habits, and eating patterns. Quality of life (QoL) was evaluated using the EuroQoL EQ-5D system. The EQ-5D index measures health status across five dimensions: mobility, self-care, usual activities, pain/discomfort, and anxiety/depression. A score closer to 1 indicates a higher quality of life. We also investigated the prevalence of anxiety and depression^19^.

Drinking was operationally defined as the consumption of alcohol at least once per month over the course of the past year, irrespective of alcohol type or quantity. Regular exercise was operationally defined as engaging in vigorous or moderate physical activity. Vigorous exercise involved high-intensity activities, such as running, for ≥1.25 hours per week. Moderate exercise involved engaging in activities at a moderate intensity, such as jogging, for ≥2.5 hours per week^19^.

### Statistical analysis

The data analyses were performed using SAS version 9.4 (SAS Institute Inc., Cary, NC, USA). Data sourced from the KNHANES were obtained using stratified and multistage clustered probability sampling methods, ensuring representation of the entire Korean population. Therefore, population weights were applied to the analyses^14^. Statistical significance was determined by a p<0.05 or a 95% confidence interval (CI) that did not span 1.0. The data are presented as mean ± standard error (SE) for continuous variables and percentage ± SE for categorical variables.

**Figure 2:**
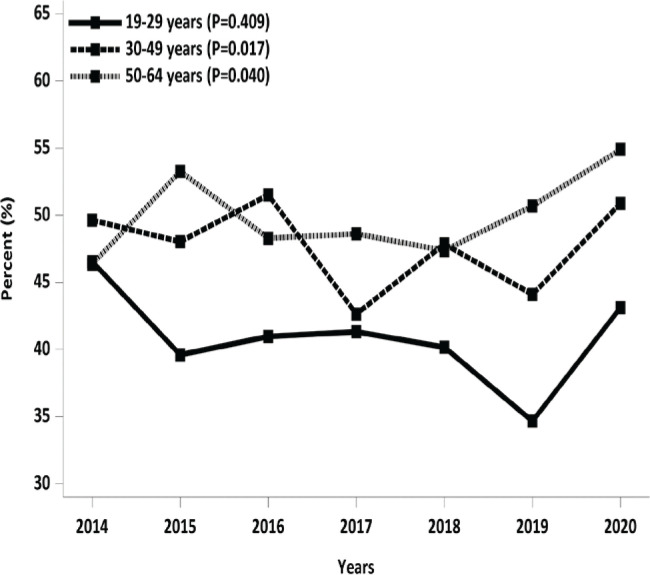
Yearly changes in perceived body image perception of obesity by age group

The Rao-Scott chi-squared test was conducted to assess the differences in perceived body image based on smoking status (Table 1). Yearly changes in perceived body image perception of obesity by age group were also examined using the Rao-Scott chi-squared test (Figure 2). Differences in sociodemographic and clinical characteristics were evaluated using analysis of variance (ANOVA) and the Rao-Scott chi-squared test (Table 2). Univariable and multivariable logistic regression analyses were performed to investigate the relationship between the body image perception of obesity and smoking status (Table 3). In the multivariable logistic regression analysis, variables such as marital status, income, education level, systolic/diastolic blood pressure, BMI, total cholesterol, hemoglobin, fasting blood sugar, trauma history, drinking rate, stress perception, anxiety/depression rate, and frequency of skipping meals (breakfast, lunch, and dinner) were controlled for.

**Table 1:** Perception of body image types by age group according to smoking status (N=12515)

	*Current smoker (CS)* *(N=742; 6.5%)*		*Hidden smoker (HS)* *(N=132; 1.0%)*		*Non-smoker (NS)* *(N=11641; 92.4%)*		*p*	*p by comparison*
	*n (wt’d n)*	*% (SE)*	*n (wt’d n)*	*% (SE)*	*n (wt’d n)*	*% (SE)*		*CS vs NS*
**Age 19–29 years** (N=1690; 19.2%)	152 (254229)		14 (19549)		1524 (2387253)		<0.001	<0.001
Thin and normal	67	43.2 (4.36)	7	50.9 (13.77)	927	60.9 (1.37)		
Obese	85	56.8 (4.36)	7	49.1 (13.77)	597	39.1 (1.37)		
**Age 30–49 years** (N=5584; 44.7%)	377 (446588)		93 (103119)		5114 (5632979)		0.008	0.003
Thin and normal	165	44.5 (2.78)	43	45.5 (5.90)	2670	52.9 (0.82)		
Obese	212	55.5 (2.78)	50	54.5 (5.90)	2444	47.1 (0.82)		
**Age 50–64 years** (N=5241; 36.1%)	213 (200155)		25 (20651)		5003 (4766934)		0.938	0.782
Thin and normal	104	48.9 (3.97)	11	52.3 (10.72)	2458	50.0 (0.85)		
Obese	109	51.1 (3.97)	14	47.7 (10.72)	2545	50.0 (0.85)		

Values are presented as sample numbers (n), weighted n, and weighted percentages (standard error, SE). The p was determined using the Rao-Scott chi-squared test. wt’d: weighted.

**Table 2:** Sociodemographic and clinical characteristics, health behaviors, and perceived health status of participants (N=12515)

	*Age 19–29 years*				*Age 30–49 years*				*Age 50–64 years*			
	*Current smoker (CS)*	*Hidden smoker (HS)*	*Non-smoker (NS)*	*p*	*Current smoker (CS)*	*Hidden smoker (HS)*	*Non-smoker (NS)*	*p*	*Current smoker (CS)*	*Hidden smoker (HS)*	*Non-smoker (NS)*	*p*
**Age** (years)	24.54 ± 0.26	24.99 ± 0.63	23.94 ± 0.09	0.026	39.60 ± 0.34	40.44 ± 0.62	40.30 ± 0.11	0.135	56.26 ± 0.35	56.39 ± 0.82	56.57 ± 0.07	0.672
**Married**	19.2 (3.48)	25.5 (12.75)	12.9 (1.07)	0.063	67.4 (2.93)	89.8 (3.14)	85.9 (0.62)	<0.001	57.6 (4.14)	50.4 (10.78)	84.4 (0.64)	<0.001
**Residence**				0.959				0.541				0.174
City	92.1 (2.37)	90.2 (6.83)	92.3 (0.94)		87.7 (2.04)	86.0 (3.75)	89.0 (0.99)		81.1 (3.19)	85.8 (6.79)	85.8 (1.00)	
Rural	7.9 (2.37)	9.8 (6.83)	7.7 (0.94)		12.3 (2.04)	14.0 (3.75)	11.0 (0.99)		18.9 (3.19)	14.2 (6.79)	14.2 (1.00)	
**Currently working**				1.581				0.363				
Yes	63.6 (4.35)	60.0 (13.72)	59.0 (1.36)		63.8 (2.78)	59.1 (6.10)	59.6 (0.86)		56.3 (4.64)	43.7 (11.39)	56.3 (0.91)	0.637
No	36.4 (4.35)	40.0 (13.72)	41.0 (1.36)		36.2 (2.78)	40.9 (6.10)	40.4 (0.86)		43.7 (4.64)	56.3 (11.39)	43.7 (0.91)	
**Household income** (quartiles)				<0.001				<0.001				<0.001
1st (lowest)	43.0 (4.62)	33.3 (13.55)	24.4 (1.42)		36.2 (2.89)	39.8 (5.55)	22.7 (0.75)		43.8 (3.98)	48.1 (10.73)	21.5 (0.72)	
2nd	22.6 (3.84)	32.1 (12.51)	24.4 (1.30)		28.9 (2.56)	16.1 (4.43)	24.3 (0.71)		27.0 (3.39)	27.4 (9.86)	24.3 (0.77)	
3rd	22.0 (3.87)	21.8 (11.25)	25.1 (1.36)		23.2 (2.58)	18.4 (4.02)	25.7 (0.72)		16.7 (3.07)	9.0 (6.08)	26.4 (0.77)	
4th (highest)	12.4 (2.93)	12.8 (8.58)	26.0 (1.33)		11.7 (1.76)	25.6 (5.12)	27.2 (0.87)		12.6 (3.05)	15.5 (8.16)	27.9 (0.92)	
**Types of health insurance**				<0.001				<0.001				<0.001
National Health Insurance	92.6 (2.45)	91.1 (8.43)	97.9 (0.42)		93.5 (1.52)	95.5 (2.29)	98.1 (0.23)		83.7 (3.32)	90.6 (6.04)	98.0 (0.25)	
Government medical aid for low income	7.4 (2.45)	8.9 (8.43)	2.1 (0.42)		6.5 (1.52)	4.5 (2.29)	1.9 (0.23)		16.3 (3.32)	9.4 (6.04)	2.0 (0.25)	
**Education level**				0.018				<0.001				0.001
≤High school	58.5 (4.56)	54.2 (13.55)	46.0 (1.46)		69.5 (2.79)	61.7 (5.64)	35.4 (0.91)		88.1 (2.76)	90.1 (6.65)	76.4 (0.88)	
University or higher	41.5 (4.56)	45.8 (13.55)	54.0 (1.46)		30.5 (2.79)	38.3 (5.64)	64.6 (0.91)		11.9 (2.76)	9.9 (6.65)	23.6 (0.88)	
**Health status**												
Systolic blood pressure (mmHg)	106.25 ± 0.82	105.11 ± 2.86	104.64 ± 0.26	0.154	110.26 ± 0.85	106.32 ± 1.58	108.35 ± 0.22	0.035	119.14 ± 1.33	116.88 ± 3.60	119.51 ± 0.29	0.736
Diastolic blood pressure (mmHg)	70.92 ± 0.69	68.27 ± 2.29	69.13 ± 0.23	0.036	74.02 ± 0.63	71.43 ± 1.23	72.62 ± 0.15	0.058	76.54 ± 0.78	75.63 ± 2.21	76.47 ± 0.16	0.927
Body mass index (kg/m^2^)	22.27 ± 0.32	23.26 ± 1.36	21.70 ± 0.11	0.141	23.47 ± 0.25	22.97 ± 0.40	22.87 ± 0.06	0.065	23.78 ± 0.27	22.83 ± 0.73	23.84 ± 0.06	0.384
Waist circumference (cm)	74.37 ± 0.77	76.23 ± 2.28	72.00 ± 0.26	0.003	78.95 ± 0.69	77.29 ± 0.97	76.81 ± 0.16	0.010	81.49 ± 0.77	79.71 ± 2.15	80.45 ± 0.16	0.395
Abdominal obesity	12.4 (2.64)	8.9 (8.43)	8.1 (0.76)	0.202	28.5 (2.66)	18.9 (4.95)	17.1 (0.63)	<0.001	33.8 (4.00)	23.1 (8.57)	27.9 (0.79)	0.221
Total cholesterol (mg/dL)	177.23 ± 2.52	174.39 ± 9.28	178.76 ± 0.85	0.766	188.56 ± 1.91	188.15 ± 3.97	190.71 ± 0.52	0.457	205.33 ± 3.92	205.41 ± 9.07	203.59 ± 0.65	0.890
Fasting blood sugar (mg/dL)	90.09 ± 0.69	89.82 ± 1.88	88.74 ± 0.39	0.212	96.51 ± 1.03	92.63 ± 1.06	93.70 ± 0.26	0.017	103.03 ± 1.57	101.47 ± 3.06	100.55 ± 0.36	0.294
Trauma history	14.9 (3.13)	18.0 (11.69)	7.0 (0.74)	0.003	7.6 (1.72)	18.1 (4.92)	4.8 (0.33)	<0.001	5.8 (1.73)	26.2 (10.39)	7.8 (0.46)	0.009
Drinking	83.2 (3.36)	72.3 (12.11)	57.7 (1.45)	<0.001	73.0 (2.76)	71.6 (5.01)	50.6 (0.81)	<0.001	60.3 (4.03)	60.9 (10.45)	34.2 (0.82)	<0.001
Participation in aerobic physical activity	56.6 (4.29)	55.1 (13.86)	58.9 (1.44)	0.838	44.7 (2.84)	50.2 (5.78)	47.7 (0.84)	0.519	32.1 (3.99)	28.9 (10.35)	45.9 (0.85)	0.001
**Perceived health status**				<0.001				<0.001				<0.001
Very good/good	19.1 (3.25)	25.8 (11.51)	39.4 (1.38)		21.0 (2.45)	17.0 (3.97)	33.6 (0.75)		15.4 (2.92)	6.2 (5.97)	24.3 (0.73)	
Fair	57.5 (4.02)	31.7 (12.23)	48.8 (1.51)		56.2 (3.01)	64.5 (5.47)	53.8 (0.77)		46.2 (4.15)	45.4 (11.32)	55.6 (0.87)	
Poor/very poor	23.3 (3.58)	42.4 (13.87)	11.8 (0.92)		22.8 (2.42)	18.6 (4.32)	12.5 (0.54)		38.4 (4.22)	48.4 (11.45)	20.1 (0.69)	
**Perceived psychological stress**	55.9 (4.20)	59.4 (13.41)	38.9 (1.34)	<0.001	48.6 (2.83)	39.3 (5.83)	28.0 (0.71)	<0.001	41.8 (3.92)	42.9 (10.68)	22.8 (0.69)	<0.001
**EuroQol: anxiety/depression**	16.5 (3.34)	19.5 (10.38)	9.1 (0.83)	0.009	17.5 (2.25)	8.6 (3.37)	6.4 (0.39)	<0.001	27.0 (4.36)	40.2 (11.46)	11.2 (0.52)	<0.001
**EQ-5D index**	0.963 ± 0.005	0.954 ± 0.020	0.978 ± 0.001	0.017	0.953 ± 0.005	0.968 ± 0.007	0.973 ± 0.001	<0.001	0.899 ± 0.013	0.914 ± 0.023	0.949 ± 0.001	<0.001

Values are presented either as weighted mean ± standard error (SE) or weighted percentage (SE). The p was determined using analysis of variance or the Rao-Scott chi-squared test. Married: living with a spouse or cohabitant.

**Table 3:** Relationship between perception of body image on obesity and smoking status by age group (N=12515)

	*Univariable logistic regression*						*Multivariable logistic regression*					
	*19–29 years*		*30–49 years*		*50 –64 years*		*19–29 years*		*30–49 years*		*50–64 years*	
	*OR (95% CI)*	*p*	*OR (95% CI)*	*p*	*OR (95% CI)*	*p*	*AOR (95% CI)*	*p*	*AOR (95% CI)*	*p*	*AOR (95% CI)*	*p*
Current smoker (CS)	2.05 (1.41–2.97)	<0.001	1.40 (1.11–1.75)	0.004	1.05 (0.76–1.44)	0.782	2.60 (1.49–4.52)	0.001	1.32 (0.89–1.96)	0.161	1.02 (0.54–1.96)	0.942
Hidden smoker (HS)	1.50 (0.51–4.46)	0.462	1.34 (0.84–2.16)	0.220	0.91 (0.39–2.12)	0.832	0.76 (0.19–3.12)	0.705	1.64 (0.85–3.17)	0.142	2.27 (0.64–8.01)	0.202
Non- smoker (NS) ®	1		1		1		1		1		1	

Multivariable logistic regression: Adjusted for marital status, income, education level, systolic/diastolic blood pressure, BMI, total cholesterol, hemoglobin, fasting blood sugar, trauma history, drinking rate, stress perception, anxiety/depression rate, and frequency of skipping meals (breakfast, lunch, and dinner). AOR: adjusted odds ratio.

® Reference category.

Significant differences were observed in women aged 19–29 years in the multivariable logistic regression analysis, and a multiple logistic regression analysis was conducted to explore the factors influencing the perception of being obese (Table 4). The following factors were controlled for in the analysis: marital status, household income, education level, systolic blood pressure, diastolic blood pressure, body mass index, total cholesterol, hemoglobin, fasting blood sugar, trauma history, drinking, perceived psychological stress, EuroQol: anxiety/depression, and skipping meals (breakfast, lunch, and dinner). The multiple logistic regression analysis used variables with p<0.1 in Table 2. All tests were two-tailed, allowing the statistical tests to detect differences or effects in both directions.

**Table 4:** Factors affecting the perception of being obese in women aged 19–29 years (N=1690)

*Variables*	*Multiple logistic regression*	
	*AOR (95% CI)*	*p*
**Household income** (quartiles)		
1st (lowest)	1.38 (0.81–2.35)	0.236
2nd	1.09 (0.66–1.82)	0.726
3rd	1.66 (1.02–2.69)	0.040
4th (highest) ®	1	
**Types of health insurance**		
National Health Insurance ®	1	
Medical aid	0.53 (0.16–1.76)	0.299
**Education level**		
≤High school ®	1	
University or higher	0.94 (0.66–1.34)	0.721
**Smoking status**		
Current smoker	2.32(1.28–4.23)	0.006
Hidden smoker	0.76(0.14–4.15)	0.756
Non-smoker ®	1	
**Health status**		
Systolic blood pressure (each 10 mmHg increase)	1.19 (0.92–1.53)	0.182
Diastolic blood pressure (each 10 mmHg increase)	0.78 (0.58–1.06)	0.120
Body mass index (kg/m^2^)	2.95 (2.59–3.37)	<0.001
Total cholesterol (each 10 mg/dL increase)	1.00 (0.94–1.07)	0.989
Triglyceride (each 10 mg/dL increase)	0.97 (0.93–1.01)	0.135
FBS (each 10 mg/dL increase)	1.03 (0.86–1.23)	0.740
Aerobic physical activity (No)	0.73(0.51–1.05)	0.089
**Perceived health status**		
Very good/good ®	1	
Fair	2.48 (1.71–3.60)	<0.001
Poor/very poor	3.82 (2.11–6.92)	<0.001
**Perceived psychological stress**	1.25 (0.86–1.82)	0.240
**EuroQol: anxiety/depression**	1.02 (0.50–2.06)	0.963

Multiple logistic regression analysis was adjusted for marital status, household income, education level, systolic blood pressure, diastolic blood pressure, body mass index, total cholesterol, hemoglobin, fasting blood sugar, trauma history, drinking, perceived psychological stress, EuroQol: anxiety/depression, and skipping meals (breakfast, lunch, and dinner). Hypertension and diabetes mellitus were excluded from the analysis because of multicollinearity. AOR: adjusted odds ratio.

® Reference categories.

## RESULTS

### Changes in subjective body image perception of obesity by year according to age group

Figure 2 illustrates the trend changes in the subjective body image perception of obesity over the years across the three age groups. In the age group of 19–29 years, there were no statistically significant yearly trend changes. However, in the age groups of 30–49 and 50–64 years, the annual trend changes were found to be statistically significant. Specifically, within the age group of 30–49 years, the percentage of individuals who perceived their body type as obese decreased from 47.9% in 2018 to 44.1% in 2019 and then increased to 50.9% in 2020 (p<0.017). In the 50–64 years age group, the percentage of individuals who perceived their body type as obese increased from 47.4% in 2018 to 54.9% in 2020 (p<0.040).

**Figure 3:**
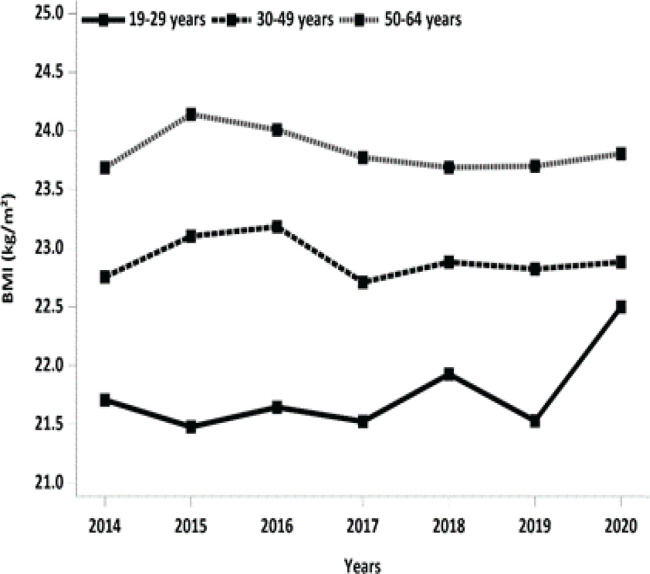
Yearly changes in mean body mass indices by age group

When comparing these findings to the annual trend changes in mean body mass indices (BMIs) across the three different age groups (Figure 3), we observed a potential disparity between actual body weight changes and changes in body image perception. For instance, contrary to the increase in body image perception of obesity, the mean BMIs in the age groups of 30–49 and 50–64 years remained stable in 2020.

### Perception of body image types by age group according to smoking status

Table 1 presents the perception of body image type by age group according to smoking status. Among women aged 19–29 years, the rates of perceiving their body type as normal or thin were 60.9%, 50.9%, and 43.2% in the NS, HS, and CS groups, respectively. Conversely, the rates of perceiving one’s body shape as obese were 39.1%, 49.1%, and 56.8% in the NS, HS, and CS groups, respectively (p<0.001). Therefore, the highest rate of perceiving body image as obese was observed in the CS group, followed by the HS group.

Among women aged 30–49 years, the rates of perceiving their body type as normal or thin were 52.9%, 45.5%, and 44.5% in the NS, HS, and CS groups, respectively. In contrast, the rates of perceiving their body type as obese were 47.1%, 54.5%, and 55.5% in the NS, HS, and CS groups, respectively (p<0.008). Thus, the highest rate of obesity perception was observed in the CS group, followed by the HS group.

No significant differences were observed in the age group of 50–64 years. Notably, there were no significant differences in mean BMIs according to smoking status in any age group (Table 2).

### Sociodemographic and clinical characteristics and health behaviors, and perceived health status of participants

Table 2 presents representative variables related to participants’ sociodemographic and health characteristics. The full version of the table, including all variables, is displayed in the Supplementary file Tables 1 and 2. Below, we highlight a few important statistically significant characteristics in each age group.

Across all age groups, lower levels of income and education level and a higher prevalence of government medical aid recipients were observed among individuals in the smoking group, either CS or HS, indicating a low socioeconomic status in these groups. In the age group of 50–64 years, being married was notably more common in the NS group.

As stated earlier, there were no significant differences in mean BMIs according to smoking status in any age group. However, waist circumferences were notably higher in the smoking groups, both CS and HS, among women aged 19–29 and 30–49 years. Nonetheless, the difference in abdominal obesity was remarkable only in the age group of 30–49 years, with the highest prevalence being 28.5% in the CS group.

Another noteworthy finding was that the prevalence of trauma history was the highest in the HS group across all age groups. Additionally, drinking prevalence and perceived psychological stress, anxiety, and depression levels were the lowest in the NS group across different age groups. Unsurprisingly, perceived health status and quality of life, assessed using the EQ-5D index, were the highest in the NS group across all age groups (Table 2).

### Relationship between perception of body image on obesity and smoking status by age group

Table 3 presents the relationship between body image perception of obesity and smoking status across the three age groups. Univariable and multivariable logistic regression analyses were performed to investigate this association. In the univariable logistic regression analysis, women aged 19–29 years in the CS group were 2.05 times more likely than those in the NS group to perceive their body type as obese (95% CI: 1.41–2.97). Similarly, women in the CS group aged 30–49 years were 1.40 times more likely than those in the NS group to perceive their body shape as obese (95% CI: 1.11–1.75). No significant relationships were observed in the 50–64 years age group.

In the subsequent multivariate logistic regression analysis, only women aged 19–29 years in the CS group were 2.60 times more likely than those in the NS group to perceive their body image as obese (95% CI: 1.49–4.52). This analysis controlled for variables such as marital status, income, education level, systolic/diastolic blood pressure, BMI, total cholesterol, hemoglobin, fasting blood sugar, trauma history, drinking rate, stress perception, anxiety/depression rate, and frequency of skipping meals (breakfast, lunch, and dinner).

### Factors affecting the perception of being obese in women aged 19–29 years

In the multivariate logistic regression analysis, only women aged 19–29 years in the CS group were more likely to perceive themselves as obese than those in the NS group (Table 3). Consequently, the factors influencing obesity perception in this age group alone were examined using multiple logistic regression analysis, as presented in Table 4. Household income, current smoking status, BMI, and perceived health status were found to be strongly associated with the perception of one’s body shape as obese in this age group.

Specifically, current smoking (AOR=2.32; 95% CI: 1.28 –4.23), higher BMI (AOR=2.95; 95% CI: 2.59–3.37), and perceiving health status as fair or poor (AOR=3.82; 95% CI: 2.11–6.92) were significant factors associated with the perception of being obese in this age group. Additionally, women in the 3rd quartile household income were 1.66 times more likely than those in the 4th quartile household income (highest quartile) to perceive their body image as obese (95% CI: 1.02–2.69). This analysis controlled for marital status, household income, education level, systolic blood pressure, diastolic blood pressure, body mass index, total cholesterol, hemoglobin, fasting blood sugar, trauma history, drinking, perceived psychological stress, EuroQol: anxiety/depression, and skipping meals (breakfast, lunch, and dinner).

## DISCUSSION

This study aimed to examine the relationship between the perception of obesity and smoking status in women according to three age groups: 19–29, 30–49, and 50–64 years.

First, we examined the perception of body image in different age groups according to smoking status. The results showed that the highest rate of obesity perception was observed in the CS group, followed by the HS group in women aged 19–29 and 30–49 years. No significant differences were observed in the 50–64 age group. Therefore, a considerably higher proportion of individuals who perceived themselves as obese among smokers than among non-smokers among women aged 19–49 years was observed. Intriguingly, there were no significant differences in mean BMIs according to smoking status in any age group. In addition, when comparing the annual trend changes in subjective body image perception of obesity to those in mean BMIs among different age groups, we observed a potential disparity between mean BMIs and changes in body image perception. Taken together, our results suggest that there may be discrepancies between the subjective perception of obesity and the actual presence of obesity among participants.

Second, as previously reported, we observed a low socioeconomic status among smokers, whether current or hidden smokers, across all age groups. Furthermore, the prevalence of alcohol consumption and perceived psychological stress, anxiety, and depression levels were significantly higher among smokers. Additionally, the perceived health status and quality of life were notably lower among smokers^3,20,21^.

Third, when investigating the relationship between the perception of body image regarding obesity and smoking status across the three age groups using multivariate logistic regression analyses, we observed that only women aged 19–29 years in the CS group were more likely to perceive their body image as obese than those in the NS group. This suggests that the perception of obesity may be a critical contributing factor to the continuation or initiation of smoking, particularly among women aged 19–29 years.

Fourth, when we further explored the factors influencing the perception of obesity in women aged 19–29 years, we identified current smoking status, higher BMI, and perceived poor health status as significant factors. The perception of obesity may contribute to a poor perceived health status. Therefore, these findings further support the association between the perception of obesity and current smoking in this age group.

Many studies have investigated the effect of smoking on body weight. Smoking is generally associated with weight loss^2-4^. For example, Wang^3^, using a national cohort, reported that cigarette smoking increased the likelihood of being underweight by 2.7% and having a healthy weight by 12.7%. Conversely, it decreased the likelihood of being overweight or obese by 13%, with obesity specifically reduced by 10% in the Chinese adult population^3^. However, the association between smoking and weight loss remains debatable^5,6^. Nonetheless, the most extensively researched aspect of the relationship between body weight and smoking, with substantial evidence, is that quitting smoking leads to weight gain, at least temporarily^2,7-9^. According to one study using national data from adults aged 25–74 years, quitting smoking was associated with a mean weight gain of 2.8 kg in men and 3.8 kg in women, even after adjusting for various factors, such as age, race, education level, alcohol use, illnesses related to weight change, baseline weight, and physical activity^2^. The relative risk of marked weight gain among quitters compared with continuing smokers was 8.1 in men and 5.8 in women, and this risk remained high irrespective of the duration of smoking cessation^2^. Furthermore, a population-based cohort study and systematic review reported that the incidence of diabetes and hypertension increased, especially within three years of quitting, among those who quit due to post-cessation weight gain^22-24^.

The proposed mechanism of smoking-induced weight reduction can be explained in two ways. First, nicotine increases the metabolic rate, thereby enhancing energy consumption. Second, it suppresses appetite. The increase in the metabolic rate is attributed to the activation of lipoprotein lipase, which breaks down triglycerides to form free fatty acids, and the activation of the sympathetic nervous system. Appetite suppression is reported to be caused by the direct stimulation of melanocortin receptor 4 (MC4-R) and increased serum levels of leptin^4,25-28^.

As the public becomes increasingly aware of the relationship between smoking and weight, numerous studies have reported that many individuals tend to smoke to control their weight or avoid quitting smoking because of fear of weight gain. These studies suggest that the effect of smoking on weight management varies with age and sex. For example, one study reported that young adults trying to lose weight were 40% more likely to smoke cigarettes^10^. Fang et al.^11^ found that the association between weight and smoking was more pronounced in females, particularly in female adolescents. This study also observed that adolescent BMI was strongly associated with increased cigarette smoking in adulthood among women^11^.

When examining the relationship between smoking and weight, it has been reported that not only a higher BMI but also merely perceiving oneself as overweight can increase smoking rates. For instance, one study found that the perceived importance of being thin among young female adolescents predicts future smoking initiation^12^. Another study reported that compared to adolescents who perceived themselves to be of the right weight or underweight, those who perceived themselves to be very overweight were 6.1 percentage points (pps) more likely to smoke currently and 3.3 pps more likely to smoke frequently. Adolescents with a slightly overweight perception are 7.9 pps more likely to smoke currently and 2.5 pps more likely to smoke frequently than those with the right weight or underweight perception. These relationships are more pronounced in females and appear to be mediated by weight-loss activities^13^.

Collectively, these studies suggest that the perception of being overweight can strongly influence smoking initiation and continuation, particularly among young females. Furthermore, this implies that perception, including the misperception of being obese or overweight rather than BMI, is highly likely to determine smoking-related behavior in young females.

In this context, we investigated the relationship between the perception of obesity, not BMI, and smoking among adult women in Korea. Unlike other studies primarily targeting adolescents, our study included all adult women. We systematically examined the relationship between smoking status and obesity perception by dividing the participants into three age groups. We found that only women aged 19–29 years in the CS group were considerably more likely to perceive themselves as obese than those in the NS group. We further observed that both current smoking and high BMI contributed to the perception of obesity in this age group. Overall, our findings align with those of previous research, showing that the perception of being overweight considerably influences young females to smoke. This study provides further solid evidence of the relationship between current smoking, high BMI, and obesity perception in young females by analyzing the factors that influence obesity perception. These findings suggest that interventions aimed at weight reduction or modifying the perception of obesity could aid smoking cessation efforts in young women.

Unlike previous studies, this study identified and analyzed hidden smokers separately from current smokers by measuring their urinary cotinine levels. This approach was adopted because Asian women often conceal their smoking status^29,30^. Classifying these individuals as non-smokers can potentially affect the analysis. Thus, we attempted to obtain more accurate results by distinguishing hidden smokers.

### Limitations

One limitation of this study stems from its cross-sectional design, specifically the inability to clearly determine the temporal relationship and mechanisms between subjective body image perception and smoking status, thereby allowing only the inference of a correlation. Additionally, if reasons for starting smoking or being unable to quit are related to weight control, detailed information on smoking behavior, such as the amount of smoking or past quitting attempts, may reveal different aspects of smokers who smoke for weight control purposes. However, due to the lack of such information, further analyses were impossible. Further, to improve the generalizability of our findings, additional research is needed to determine whether the results can be replicated in other cultures, as perceptions of obesity may vary across different cultural contexts. In addition, despite our efforts to adjust for potential confounding variables, residual confounding may persist owing to unmeasured or inadequately measured factors.

## CONCLUSIONS

This study examined the relationship between body image perception and smoking status among women aged 19–64 years in Korea. This study identified a notable relationship between the perception of obesity and smoking among women aged 19–29 years only. We also found that both current smoking and a high BMI were factors influencing the perception of obesity in this age group.

## Supplementary Material



## Data Availability

The data supporting this research are available from the authors on reasonable request.
