# Supplementary material for: Relationship between perception of body image on obesity and smoking status by age group in women: Findings of a seven-year Korean National Survey
Source: Tob Induc Dis. 2024 Oct 25;22:10.18332/tid/194098. doi: 10.18332/tid/194098 (PMC11504196; doi:10.18332/tid/194098)
Supplement: Supplementary file 1 [file TID-22-170-s1.pdf]

Supplementary Materials

Relationship between perception of body image on obesity and smoking status according to age groups in women: Findings of a 7-year Korea National Survey

Hye Jung Hwang, Youngmee Kim, And Won-Kyung Cho

Supplementary Table 1. Sociodemographic characteristics of participants (N=12,515)

Age Group: 19–29 years

	Current smoker (CS)	Hidden smoker (HS)	Non-smoker (NS)	*p*	*p* by multiple comparison: CS vs. HS	CS vs. NS	HS vs. NS
Age (year)	24.54 ± 0.26	24.99 ± 0.63	23.94 ± 0.09	0.026	0.511	0.031	0.094
Married (%)	19.2 (3.48)	25.5 (12.75)	12.9 (1.07)	0.063	0.609	0.048	0.204
Residence (%)				0.959	0.767	0.946	0.745
City	92.1 (2.37)	90.2 (6.83)	92.3 (0.94)				
Rural	7.9 (2.37)	9.8 (6.83)	7.7 (0.94)				
Economic activity (%)				1.581	0.801	0.331	0.940
Yes	63.6 (4.35)	60.0 (13.72)	59.0 (1.36)				
No	36.4 (4.35)	40.0 (13.72)	41.0 (1.36)				
Household income (Quartiles)				<0.001	0.858	<.001	0.626
1st	43.0	33.3	24.4				

Age Group: 30–49 years

	Current smoker (CS)	Hidden smoker (HS)	Non-smoker (NS)	*p*	*p* by multiple comparison: CS vs. HS	CS vs. NS	HS vs. NS
Age (year)	39.60 ± 0.34	40.44 ± 0.62	40.30 ± 0.11	0.135	0.220	0.051	0.819
Married (%)	67.4 (2.93)	89.8 (3.14)	85.9 (0.62)	<0.001	<0.001	<0.001	0.286
Residence (%)				0.541	0.647	0.485	0.368
City	87.7 (2.04)	86.0 (3.75)	89.0 (0.99)				
Rural	12.3 (2.04)	14.0 (3.75)	11.0 (0.99)				
Economic activity (%)				0.363	0.480	0.150	0.938
Yes	63.8 (2.78)	59.1 (6.10)	59.6 (0.86)				
No	36.2 (2.78)	40.9 (6.10)	40.4 (0.86)				
Household income (Quartiles)				<0.001	0.004	<0.001	0.004
1st	36.2	39.8	22.7				

Age Group: 50–64 years

	Current smoker (CS)	Hidden smoker (HS)	Non-smoker (NS)	*p*	*p* by multiple comparison: CS vs. HS	CS vs. NS	HS vs. NS
Age (year)	56.26 ± 0.35	56.39 ± 0.82	56.57 ± 0.07	0.672	0.882	0.386	0.829
Married (%)	57.6 (4.14)	50.4 (10.78)	84.4 (0.64)	<0.001	0.520	<0.001	<0.001
Residence (%)				0.174	0.559	0.091	0.999
City	81.1 (3.19)	85.8 (6.79)	85.8 (1.00)				
Rural	18.9 (3.19)	14.2 (6.79)	14.2 (1.00)				
Economic activity (%)							
Yes	56.3 (4.64)	43.7 (11.39)	56.3 (0.91)	0.637	0.301	0.999	0.270
No	43.7 (4.64)	56.3 (11.39)	43.7 (0.91)				
Household income (Quartiles)				<0.001	0.827	<0.001	0.015
1st	43.8	48.1	21.5				

(Lowest)	(4.62)	(13.55)	(1.42)					(2.89)	(5.55)	(0.75)					(3.98)	(10.73)	(0.72)				
2nd	22.6 (3.84)	32.1 (12.51)	24.4 (1.30)					28.9 (2.56)	16.1 (4.43)	24.3 (0.71)					27.0 (3.39)	27.4 (9.86)	24.3 (0.77)				
3rd	22.0 (3.87)	21.8 (11.25)	25.1 (1.36)					23.2 (2.58)	18.4 (4.02)	25.7 (0.72)					16.7 (3.07)	9.0 (6.08)	26.4 (0.77)				
4th (Highest)	12.4 (2.93)	12.8 (8.58)	26.0 (1.33)					11.7 (1.76)	25.6 (5.12)	27.2 (0.87)					12.6 (3.05)	15.5 (8.16)	27.9 (0.92)				
Types of health insurance (%)				<0.001	0.853	<.001	0.119				<0.001	0.514	<0.001	0.080				<0.001	0.388	<0.001	0.013
National Health Insurance	92.6 (2.45)	91.1 (8.43)	97.9 (0.42)					93.5 (1.52)	95.5 (2.29)	98.1 (0.23)					83.7 (3.32)	90.6 (6.04)	98.0 (0.25)				
Government medical aids for low income	7.4 (2.45)	8.9 (8.43)	2.1 (0.42)					6.5 (1.52)	4.5 (2.29)	1.9 (0.23)					16.3 (3.32)	9.4 (6.04)	2.0 (0.25)				
Education level (%)				0.018	0.765	0.011	0.545				<0.001	0.205	<0.001	<0.001				0.001	0.787	0.001	0.149
≤High school	58.5 (4.56)	54.2 (13.55)	46.0 (1.46)					69.5 (2.79)	61.7 (5.64)	35.4 (0.91)					88.1 (2.76)	90.1 (6.65)	76.4 (0.88)				
University or higher	41.5 (4.56)	45.8 (13.55)	54.0 (1.46)					30.5 (2.79)	38.3 (5.64)	64.6 (0.91)					11.9 (2.76)	9.9 (6.65)	23.6 (0.88)				

Values are presented as weighted mean ± standard error (SE) or weighted percentage (SE). The *p* value was determined using analysis of variance –or the Rao-Scott Chi-Square test.

Supplementary Table 2. Clinical characteristics, health behaviours, and perceived health status of participants (N=12,515)

	19–29 years old							30–49 years old							50–64 years old						
	Current smoker (CS)	Hidden smoker (HS)	Non-smoker (NS)	*p*	*p* by multiple comparison			Current smoker (CS)	Hidden smoker (HS)	Non-smoker (NS)	*p*	*p* by multiple comparison			Current smoker (CS)	Hidden smoker (HS)	Non-smoker (NS)	*p*	*p* by multiple comparison		
					CS vs. HS	CS vs. NS	HS vs. NS					CS vs. HS	CS vs. NS	HS vs. NS					CS vs. HS	CS vs. NS	HS vs. NS
SBP (mmHg)	106.25 ± 0.82	105.11 ± 2.86	104.64 ± 0.26	0.154	0.701	0.053	0.870	110.26 ± 0.85	106.32 ± 1.58	108.35 ± 0.22	0.035	0.027	0.030	0.205	119.14 ± 1.33	116.88 ± 3.60	119.51 ± 0.29	0.736	0.557	0.783	0.464
DBP (mmHg)	70.92 ± 0.69	68.27 ± 2.29	69.13 ± 0.23	0.036	0.262	0.012	0.708	74.02 ± 0.63	71.43 ± 1.23	72.62 ± 0.15	0.058	0.060	0.031	0.338	76.54 ± 0.78	75.63 ± 2.21	76.47 ± 0.16	0.927	0.701	0.927	0.702
BMI (kg/m^2)	22.27 ± 0.32	23.26 ± 1.36	21.70 ± 0.11	0.141	0.476	0.095	0.255	23.47 ± 0.25	22.97 ± 0.40	22.87 ± 0.06	0.065	0.277	0.020	0.802	23.78 ± 0.27	22.83 ± 0.73	23.84 ± 0.06	0.384	0.221	0.829	0.169
Waist circumference (cm)	74.37 ± 0.77	76.23 ± 2.28	72.00 ± 0.26	0.003	0.442	0.004	0.066	78.95 ± 0.69	77.29 ± 0.97	76.81 ± 0.16	0.010	0.158	0.003	0.627	81.49 ± 0.77	79.71 ± 2.15	80.45 ± 0.16	0.395	0.434	0.192	0.730
abdominal obesity (%)	12.4 (2.64)	8.9 (8.43)	8.1 (0.76)	0.202	0.726	0.071	0.926	28.5 (2.66)	18.9 (4.95)	17.1 (0.63)	<.001	0.121	<.001	0.703	33.8 (4.00)	23.1 (8.57)	27.9 (0.79)	0.221	0.294	0.137	0.597
Total cholesterol (mg/dL)	177.23 ± 2.52	174.39 ± 9.28	178.76 ± 0.85	0.766	0.766	0.562	0.639	188.56 ± 1.91	188.15 ± 3.97	190.71 ± 0.52	0.457	0.927	0.276	0.522	205.33 ± 3.92	205.41 ± 9.07	203.59 ± 0.65	0.890	0.994	0.661	0.841
LDL cholesterol (mg/dL)	100.00 ± 2.55	98.47 ± 5.56	103.99 ± 0.73	0.199	0.804	0.130	0.325	109.15 ± 1.85	110.87 ± 3.76	114.35 ± 0.44	0.015	0.683	0.006	0.357	122.49 ± 3.68	123.68 ± 6.67	124.89 ± 0.58	0.801	0.876	0.520	0.857
HDL cholesterol (mg/dL)	58.86 ± 1.18	56.14 ± 3.20	59.04 ± 0.36	0.658	0.423	0.882	0.364	56.44 ± 0.75	58.59 ± 1.34	57.03 ± 0.21	0.370	0.160	0.455	0.249	53.97 ± 1.24	56.44 ± 2.50	54.49 ± 0.22	0.679	0.379	0.675	0.438
Triglyceride (mg/dL)	99.01 ± 6.88	104.81 ± 17.47	79.89 ± 1.33	0.009	0.757	0.006	0.156	124.53 ± 4.75	96.23 ± 5.60	99.81 ± 1.27	<.001	<.001	<.001	0.532	161.54 ± 11.91	125.03 ± 16.25	123.14 ± 1.25	0.006	0.071	0.001	0.908
Haemoglobin (g/dL)	13.43 ± 0.08	13.32 ± 0.33	13.15 ± 0.03	0.005	0.736	0.001	0.617	13.35 ± 0.07	13.28 ± 0.16	12.86 ± 0.02	<.001	0.706	<.001	0.009	13.68 ± 0.09	13.22 ± 0.16	13.30 ± 0.02	<.001	0.014	<.001	0.600
Fasting Blood Sugar (mg/dL)	90.09 ± 0.69	89.82 ± 1.88	88.74 ± 0.39	0.212	0.891	0.086	0.573	96.51 ± 1.03	92.63 ± 1.06	93.70 ± 0.26	0.017	0.009	0.008	0.313	103.03 ± 1.57	101.47 ± 3.06	100.55 ± 0.36	0.294	0.649	0.123	0.764

Hypertension (%)	1.1 (0.76)	-	1.6 (0.35)	-	-	0.595	-	12.5 (2.09)	8.6 (3.91)	7.0 (0.40)	0.007	0.427	0.001	0.660	33.9 (4.10)	33.9 (10.25)	30.4 (0.76)	0.609	0.995	0.395	0.723
Diabetes Mellitus (%)	0.3 (0.28)	-	0.8 (0.27)	-	-	0.318	-	6.5 (1.69)	1.3 (0.93)	3.3 (0.30)	0.007	0.016	0.016	0.169	13.9 (2.52)	16.1 (7.44)	12.8 (0.56)	0.810	0.769	0.659	0.627
Cardiovascular disease (%)	-	-	0.0 (0.03)	-				0.2 (0.16)	0.9 (0.87)	0.3 (0.08)	0.429	0.183	0.555	0.279	3.3 (1.03)	17.2 (8.05)	2.4 (0.24)	<.001	0.002	0.360	<.001
Cancer %)	0.8 (0.75)	-	0.5 (0.18)	-	-	0.659	-	1.1 (0.65)	0.9 (0.93)	3.7 (0.32)	0.019	0.894	0.027	0.129	5.2 (1.72)	8.3 (7.85)	8.6 (0.50)	0.344	0.631	0.119	0.974
Anaemia (%)	8.1 (2.23)	7.3 (7.06)	8.7 (0.79)	0.957	0.918	0.811	0.859	9.6 (1.72)	10.8 (3.55)	17.1 (0.59)	0.001	0.752	<.001	0.149	5.3 (1.94)	4.0 (3.25)	7.2 (0.47)	0.548	0.737	0.427	0.461
Arthritis, rheumatoid arthritis, or osteoporosis (%)	0.8 (0.79)	-	0.5 (0.17)	-	-	0.722	-	4.7 (1.31)	6.8 (3.41)	3.3 (0.29)	0.193	0.509	0.237	0.152	23.5 (3.34)	27.5 (9.56)	25.8 (0.71)	0.778	0.679	0.520	0.857
Liver cirrhosis (%)	-	-	-	-				-	-	0.0 (0.02)	-				-	4.4 (4.30)	0.3 (0.11)	-	-	-	<.001
Trauma History (%)	14.9 (3.13)	18.0 (11.69)	7.0 (0.74)	0.003	0.788	0.001	0.163	7.6 (1.72)	18.1 (4.92)	4.8 (0.33)	<.001	0.015	0.045	<.001	5.8 (1.73)	26.2 (10.39)	7.8 (0.46)	0.009	0.001	0.316	0.004
Low-activity participation (%)	7.9 (2.54)	6.6 (6.37)	2.8 (0.47)	0.004	0.854	0.004	0.382	5.8 (1.46)	8.4 (3.28)	3.2 (0.29)	0.006	0.440	0.024	0.015	12.4 (2.54)	11.1 (6.91)	7.3 (0.43)	0.029	0.863	0.015	0.510
Drinking (%)	83.2 (3.36)	72.3 (12.11)	57.7 (1.45)	<.001	0.318	<.001	0.279	73.0 (2.76)	71.6 (5.01)	50.6 (0.81)	<.001	0.810	<.001	<.001	60.3 (4.03)	60.9 (10.45)	34.2 (0.82)	<.001	0.958	<.001	0.009
Participation in aerobic PA (%)	56.6 (4.29)	55.1 (13.86)	58.9 (1.44)	0.838	0.920	0.608	0.784	44.7 (2.84)	50.2 (5.78)	47.7 (0.84)	0.519	0.388	0.309	0.662	32.1 (3.99)	28.9 (10.35)	45.9 (0.85)	0.001	0.783	0.001	0.136
Perceived health status (%)				<.001	0.152	<.001	0.002				<.001	0.395	<.001	0.005				<.001	0.521	<.001	0.008
Very good/Good	19.1 (3.25)	25.8 (11.51)	39.4 (1.38)					21.0 (2.45)	17.0 (3.97)	33.6 (0.75)					15.4 (2.92)	6.2 (5.97)	24.3 (0.73)				
Fair	57.5 (4.02)	31.7 (12.23)	48.8 (1.51)					56.2 (3.01)	64.5 (5.47)	53.8 (0.77)					46.2 (4.15)	45.4 (11.32)	55.6 (0.87)				
Poor/Very poor	23.3 (3.58)	42.4 (13.87)	11.8 (0.92)					22.8 (2.42)	18.6 (4.32)	12.5 (0.54)					38.4 (4.22)	48.4 (11.45)	20.1 (0.69)				
Perceived psychological stress (%)	55.9 (4.20)	59.4 (13.41)	38.9 (1.34)	<.001	0.803	<.001	0.127	48.6 (2.83)	39.3 (5.83)	28.0 (0.71)	<.001	0.162	<.001	0.038	41.8 (3.92)	42.9 (10.68)	22.8 (0.69)	<.001	0.925	<.001	0.028
EuroQol: anxiety/ depression	16.5 (3.34)	19.5 (10.38)	9.1 (0.83)	0.009	0.770	0.009	0.176	17.5 (2.25)	8.6 (3.37)	6.4 (0.39)	<.001	0.068	<.001	0.449	27.0 (4.36)	40.2 (11.46)	11.2 (0.52)	<.001	0.258	<.001	<.001

(%)																					
EQ-5D index	0.963 ± 0.005	0.954 ± 0.020	0.978 ± 0.001	0.017	0.663	0.009	0.234	0.953 ± 0.005	0.968 ± 0.007	0.973 ± 0.001	<.001	0.091	<.001	0.537	0.899 ± 0.013	0.914 ± 0.023	0.949 ± 0.001	<.001	0.582	<.001	0.125
Skipping meals (%)																					
Skipping breakfast	64.4 (4.15)	63.0 (13.27)	48.1 (1.46)	<.001	0.913	<.001	0.284	42.7 (3.01)	37.4 (5.63)	25.4 (0.74)	<.001	0.407	<.001	0.020	38.5 (3.91)	14.5 (7.99)	14.0 (0.58)	<.001	0.038	<.001	0.954
Skipping lunch	16.1 (3.46)	30.3 (12.73)	11.6 (0.86)	0.059	0.183	0.159	0.037	13.8 (1.99)	12.6 (3.64)	7.6 (0.42)	<.001	0.763	<.001	0.092	15.1 (3.01)	12.7 (7.02)	7.0 (0.41)	<.001	0.769	<.001	0.282
Skipping dinner	8.1 (2.41)	29.3 (11.85)	10.1 (0.85)	0.062	0.010	0.473	0.014	9.3 (1.77)	8.6 (4.01)	6.6 (0.42)	0.258	0.881	0.096	0.581	7.7 (2.17)	9.4 (5.69)	6.9 (0.43)	0.826	0.764	0.695	0.608
Daily calorie intake (kcal)	1891.15 ± 93.62	1339.83 ± 187.30	1799.80 ± 22.48	0.033	0.009	0.342	0.015	1759.60 ± 42.09	1779.04 ± 118.75	1754.63 ± 11.01	0.973	0.878	0.909	0.838	1604.82 ± 42.91	1398.18 ± 123.25	1687.04 ± 11.23	0.013	0.112	0.063	0.019
Daily carbohydrate intake (g)	235.83 ± 10.56	161.70 ± 25.95	252.18 ± 3.08	<.001	0.008	0.139	<.001	234.80 ± 5.33	268.27 ± 25.15	263.83 ± 1.73	<.001	0.193	<.001	0.860	251.50 ± 8.03	207.04 ± 17.19	278.50 ± 1.95	<.001	0.018	0.001	<.001
Daily protein intake (g)	67.32 ± 3.73	54.13 ± 11.05	66.40 ± 1.02	0.527	0.260	0.808	0.270	65.69 ± 1.98	62.70 ± 4.27	64.98 ± 0.51	0.815	0.527	0.723	0.598	55.08 ± 1.90	49.40 ± 6.05	59.97 ± 0.49	0.011	0.369	0.013	0.081
Daily fat intake (g)	52.20 ± 3.57	33.49 ± 8.32	52.55 ± 1.02	0.078	0.039	0.925	0.024	44.09 ± 1.64	42.34 ± 3.02	44.44 ± 0.46	0.778	0.615	0.836	0.496	33.93 ± 1.61	36.33 ± 7.50	35.28 ± 0.41	0.712	0.754	0.418	0.889
Daily calcium intake (mg)	436.40 ± 27.52	284.94 ± 64.19	456.99 ± 7.70	0.024	0.029	0.462	0.008	448.16 ± 19.01	426.51 ± 30.28	475.77 ± 4.38	0.107	0.541	0.153	0.106	442.24 ± 18.78	409.84 ± 59.74	499.26 ± 5.25	0.005	0.607	0.004	0.135
Daily iron intake (mg)	10.97 ± 0.79	8.63 ± 2.26	10.68 ± 0.18	0.623	0.331	0.721	0.365	11.21 ± 0.42	11.41 ± 0.89	12.13 ± 0.12	0.085	0.835	0.033	0.418	10.92 ± 0.51	9.52 ± 1.12	12.69 ± 0.13	<.001	0.257	<.001	0.005
Daily sodium intake (mg)	2998.22 ± 173.34	1994.21 ± 492.76	2975.87 ± 53.71	0.141	0.057	0.902	0.048	3404.94 ± 113.29	3094.49 ± 218.99	3206.26 ± 31.20	0.194	0.209	0.085	0.616	2782.01 ± 131.17	2230.55 ± 242.75	2969.81 ± 32.27	0.005	0.044	0.164	0.003
Daily Sodium intake ≥ 2000 mg/day (%)	65.2 (4.21)	22.2 (11.47)	67.3 (1.32)	0.006	0.002	0.631	<.001	77.5 (2.49)	68.2 (5.64)	73.7 (0.71)	0.203	0.108	0.157	0.304	64.9 (4.37)	41.9 (10.50)	66.8 (0.78)	0.099	0.041	0.667	0.014
Energy contribution																					
Carbohydrate (%)	53.06 ± 1.36	50.69 ± 5.00	57.51 ± 0.34	0.002	0.656	0.001	0.174	55.88 ± 0.89	60.76 ± 1.77	61.11 ± 0.19	<.001	0.015	<.001	0.841	63.43 ± 1.20	61.16 ± 2.89	66.55 ± 0.19	0.008	0.463	0.011	0.063
Protein (%)	14.65 ± 0.47	16.29 ± 2.37	14.76 ± 0.13	0.788	0.496	0.811	0.520	15.00 ± 0.34	14.30 ± 0.50	14.85 ± 0.07	0.510	0.260	0.672	0.275	13.80 ± 0.31	14.03 ± 0.65	14.27 ± 0.07	0.320	0.743	0.139	0.723
Fat (%)	24.07	21.92	25.31	0.26	0.5	0.20	0.28	22.02	20.95	22.09	0.52	0.35	0.90	0.25	18.47	21.65	18.39	0.35	0.1	0.91	0.14

	± 0.94	± 3.18	± 0.27	4	16	1	8	± 0.57	± 1.00	± 0.14	6	4	3	9	± 0.66	± 2.24	± 0.15	2	71	1	8

Values are presented as sample numbers and weighted% (standard error). The *p* value was determined using analysis of variance or the Rao-Scott Chi-Square test.
Abbreviations: SBP, systolic blood pressure; DBP, diastolic blood pressure; CVD, cardiovascular disease; PA, physical activity; LDL, low-density lipoproteins; HDL, high-density lipoproteins.
Note: CVD was defined as having a medical diagnosis of stroke, angina pectoris, or myocardial infarction. Cancer was defined as having a medical history of any type of cancer, and low levels of activity were defined as participants who responded that they were currently experiencing restrictions in daily life and social activities owing to health problems or physical or mental disabilities.

© 2024 Hwang H.J. et al.
